# Complement Dependent and Independent Interaction Between Bovine Conglutinin and *Mycobacterium bovis* BCG: Implications in Bovine Tuberculosis

**DOI:** 10.3389/fimmu.2018.03159

**Published:** 2019-02-05

**Authors:** Arshad Mehmood, Lubna Kouser, Anuvinder Kaur, Uffe Holmskov, Mohammed N. Al-Ahdal, Robert B. Sim, Uday Kishore, Anthony G. Tsolaki

**Affiliations:** ^1^Biosciences, Department of Life Sciences, College of Health and Life Sciences, Brunel University London, Uxbridge, United Kingdom; ^2^Allergy and Clinical Immunology, National Heart and Lung Institute, Imperial College London, London, United Kingdom; ^3^Department of Cancer and Inflammation, University of Southern Denmark, Odense, Denmark; ^4^Department of Infection and Immunity, King Faisal Specialist Hospital and Research Centre, Riyadh, Saudi Arabia; ^5^Department of Biochemistry, University of Oxford, Oxford, United Kingdom

**Keywords:** complement, conglutinin, mycobacterium, phagocytosis, macrophage, bovine tuberculosis, BCG, cytokine

## Abstract

Bovine conglutinin, the first animal collectin to be discovered, is structurally very similar to Surfactant Protein D (SP-D). SP-D is known to interact with *Mycobacterium tuberculosis*, and the closely-related *M. bovis*, the causative agent of bovine tuberculosis. We speculated that due to the overall similarities between conglutinin and SP-D, conglutinin is likely to have a protective influence in bovine tuberculosis. We set out to investigate the role of conglutinin in host-pathogen interaction during mycobacterial infection. We show here that a recombinant truncated form of conglutinin (rfBC), composed of the neck and C-type lectin domains, binds specifically and in a dose-dependent manner to the model organism *Mycobacterium bovis* BCG. rfBC showed a significant direct bacteriostatic effect on the growth of *M. bovis* BCG in culture. In addition, rfBC inhibited the uptake of *M. bovis* BCG by THP-1 macrophages (human monocyte lineage cell line) and suppressed the subsequent pro-inflammatory response. Conglutinin is well-known as a binder of the complement activation product, iC3b. rfBC was also able to inhibit the uptake of complement-coated *M. bovis* BCG by THP-1 macrophages, whilst modulating the pro-inflammatory response. It is likely that rfBC inhibits the phagocytosis of mycobacteria by two distinct mechanisms: firstly, rfBC interferes with mannose receptor-mediated uptake by masking lipoarabinomannan (LAM) on the mycobacterial surface. Secondly, since conglutinin binds iC3b, it can interfere with complement receptor-mediated uptake via CR3 and CR4, by masking interactions with iC3b deposited on the mycobacterial surface. rfBC was also able to modulate the downstream pro-inflammatory response in THP-1 cells, which is important for mobilizing the adaptive immune response, facilitating containment of mycobacterial infection. In conclusion, we show that conglutinin possesses complement-dependent and complement-independent anti-mycobacterial activities, interfering with both known mechanisms of mycobacterial uptake by macrophages. As mycobacteria are specialized intracellular pathogens, conglutinin may inhibit *M. bovis* and *M. tuberculosis* from establishing an intracellular niche within macrophages, and thus, negatively affect the long-term survival of the pathogen in the host.

## Introduction

Conglutinin is a C-type lectin (collectin) that is uniquely found in *Bovidae* species (e.g., cattle, *Bos taurus*) (1). Collectins are a key group of innate immune molecules which share a common overall structure, characterized by oligomerization of a polypeptide which contains a C-terminal carbohydrate recognition domain (CRD) attached to an N-terminal collagen-like region via an α-helical coiled neck region (2). Collectins are soluble pattern recognition receptors (PRR) that can recognize a variety of microbial ligands, via pathogen-associated molecular patterns (PAMPs), allergens and other host molecules leading to clearance through phagocytosis (and for some collectins, through complement activation) (3). Members of the collectin family include surfactant proteins A (SP-A) and D (SP-D), mannose binding lectin (MBL), a number of bovine collectins (conglutinin, CL-1, CL-P1, CL-43, and CL-46) and several lesser known proteins (2). Conglutinin has close structural similarities to SP-D, being able to form multivalent cruciform structures (tetramers of trimers) containing in total 12 CRD regions that can bind to microbial surfaces in the presence of Ca^2+^ (2). Conglutinin is secreted largely by the liver and is found in bovine serum at a concentration of 12 μg/ml (4). In contrast, SP-D is primarily found in the lungs at a concentration of 0.1–0.9 μg/ml and is secreted by alveolar type II cells and Clara cells (5). Conglutinin is thought to have evolved in the *Bovidae* from a gene duplication event of an ancestral SP-D gene and is located on chromosome 28 in *B. taurus* (analogous to chromosome 10 in Homo sapiens), proximal to the bovine SP-D gene (6).

The precise biological role of conglutinin is still not fully understood. Low levels of conglutinin have been observed during acute infections, such as pneumonia or metritis (7), and among some cattle breeds that are predisposed to respiratory infection (4). Like SP-D, conglutinin has been shown to bind to several microbes including viruses (e.g., Influenza A and rotavirus) (8–10), Gram-negative bacteria, such as *Escherichia coli* and *Salmonella typhimurium* (11, 12), as well as lipopolysaccharide (LPS) (13).

The complement system is a crucial first line of defense against pathogens and is composed of three pathways: classical, alternative and lectin pathway, which require different stimuli for activation. Complement activation results in the formation of a C3 convertase and the deposition of C3b on target surfaces which can trigger opsonization and other immunoregulatory functions. Conglutinin has the unique ability to bind to iC3b (a proteolytically cleaved form of complement cleavage fragment C3b), due to its specific affinity for mannose oligosaccharides on the α-chain of iC3b, which become exposed when C3b is cleaved (14). Conglutinin has been shown to bind to iC3b-coated erythrocytes, resulting in their agglutination (14, 15), to yeast mannan (16), and to iC3b-coated *E. coli*, enhancing the respiratory burst of phagocytes (11). Other collectins can also mediate interactions between microbes and phagocytes. SP-A and SP-D have been shown to bind to several bacteria, virus and fungi and influence their uptake by phagocytes (17). In contrast, the anti-microbial properties of conglutinin in this regard remain to be more fully explored. Conglutinin has been observed to strongly inhibit hemagglutination and infectivity of Influenza A virus, causing agglutination of viral particles and also to act as an opsonin, enhancing phagocytic responses against the virus (8, 18). Furthermore, conglutinin has protective activity *in vivo*, where its sub-cutaneous injection increased the survival of mice experimentally infected with highly virulent strains of *S. typhimurium* (12). Similarly, MBL, SP-D, and SP-A have also been shown to be protective *in vivo* (19).

A major infection of the bovine host is bovine tuberculosis, and, it is, therefore, of great importance to investigate what role conglutinin plays in its pathogenesis. In cattle, bovine tuberculosis accounts for substantial economic cost and presents a risk of human infection (20–23). The causative agent, *M. bovis*, can also cause infection in a wide variety of mammals including wildlife e.g., Eurasian Badgers (*Meles meles*) (22), which are thought to be a reservoir for bovine tuberculosis infection in cattle. We have previously shown that complement activation can occur on *M. bovis* BCG via the classical, alternative and lectin pathways, which results in the deposition of C3b and iC3b on the mycobacterial surface (24). Mycobacteria are specialized intracellular pathogens that have evolved to survive and persist within phagocytes and its major consequence is latent infection (25). Complement deposition on the surface of *M. tuberculosis* has also been shown to enhance its phagocytosis by macrophages and that this is mediated by complement receptors on the host cell surface (26–31). Similarly, SP-D was found to bind to *M. tuberculosis* and inhibit its uptake by macrophages via the mannose receptor on the host cell (32). We find these two observations on *M. tuberculosis* intriguing, given the known similarities of conglutinin to SP-D and the ability of conglutinin to bind iC3b.

In the present study, we have set out to investigate the role of conglutinin in *M. bovis* infection. There is very little published work on this topic. One paper reports that conglutination (erythrocyte-agglutination by conglutinin) activity in bovine serum decreased in responses to *M. bovis* infection in cattle (33). We have used a recombinant form of truncated bovine conglutinin (rfBC), composed of the α-helical neck region and the CRD of conglutinin (13) to investigate the binding of conglutinin to the vaccine strain *M. bovis* BCG (a model organism for *M. bovis*) and its effect on THP-1 macrophages (a model cell line for phagocytes). We show that rfBC binds to *M. bovis* BCG in a complement dependent as well as independent manner, and interferes with uptake of the bacterium by THP-1 cells. We also show evidence of an altered pro-inflammatory response, which is likely to influence the subsequent adaptive immune response that is crucial in tuberculosis pathogenesis. Thus, we provide the first fundamental data on the role of conglutinin in mycobacterial infection and its complement dependent and independent interactions, showing its ability to interfere with two major mechanisms of pathogen uptake by macrophages.

## Materials and Methods

### Expression and Purification of a Truncated Form of Recombinant Bovine Conglutinin (rfBC)

A recombinant polypeptide composed of the neck and the CRD regions of bovine conglutinin was expressed in *Escherichia coli*, and purified, as previously described (13). This preparation consists of amino acid residues 197–351 of the mature protein and has an expected monomer molecular weight of about 23 kDa (13). Endotoxins were removed using a polymyxin B column (Sigma-Aldrich). The levels of endotoxin in the purified protein was measured using chromogenic Limulus Amebocyte Lysate (LAL) endotoxin assay kit (GenScript), and found to be <5 pg/μg of rfBC.

Commercially available recombinant maltose-binding protein (MBP) (NEB; E8200S) was used a negative control protein.

### Western Blotting

Following 12% v/v SDS-PAGE, the gel was equilibrated in blotting buffer (12 mM Tris, 96 mM glycine, 20% methanol, pH 8.3) and transferred, using a Mini Trans-Blot Cell (Bio-Rad), onto a PVDF membrane (Thermo Fisher Scientific). The membrane was then washed in PBS and blocked with low-fat 5% milk (w/v) in PBS overnight. For immunostaining, membranes were incubated for 1 h at room temperature with either rabbit anti-bovine conglutinin IgG antibody (University of Odense, Denmark; diluted 1:1,000), or mouse anti-maltose-binding protein (MBP) monoclonal IgG antibody (NEB, E8032S; 1 μg/ml, diluted 1:10,000 in PBS, 0.05% Tween 20. Membranes were then incubated for 1 h with polyclonal goat anti-rabbit IgG conjugated to horseradish peroxidase (HRP) or rabbit anti-mouse IgG-HRP conjugate, respectively. (Sigma-Aldrich), diluted 1:1,000 in PBS, 0.05% Tween 20. Membranes were developed using Clarity Western ECL kit (BioRad) and visualized on a ChemiDoc™ XRS+ Molecular Imager (BioRad).

### Characterization of rfBC Oligomerization via Chemical Cross-Linking

To confirm that rfBC formed a homotrimeric structure, as shown in the previous study (13), a chemical cross-linking procedure was performed using a BS^3^ [bis(sulfosuccinimidyl)suberate] (Thermo Fisher Scientific). Aliquots of rfBC protein were made (100 μl of 20 μg/ml) for 8 time-point reactions (0, 1, 2, 4, 8, 16, 32, 64 min). 5 μl of 1 mM BS^3^ in dimethyl sulfoxide was then added to each aliquot and incubated at room temperature. After incubation, 1 M Tris-HCl (pH 7.5) was added to a final concentration of 20 mM to stop the reaction. Cross-linked proteins were then visualized by SDS-PAGE and Coomassie blue staining.

### Mycobacterial Cell Culture

*M. bovis* BCG (Pasteur strain; ATCC) was grown in liquid culture using Middlebrook 7H9 medium (Sigma-Aldrich), supplemented with 0.2% (v/v) glycerol, 0.05% (v/v) Tween-80, and 10% (v/v) albumin-dextrose-catalase (ADC) (BD BBL, Becton Dickinson). Green fluorescent protein (GFP)-expressing *M. bovis* BCG (Danish Strain 1331) containing the pGFPHYG2 plasmid (a gift from Dr. B. Robertson, Imperial College London, UK) was grown in the above medium but with the addition of 50 μg/ml of hygromycin to maintain the plasmid. Cultures were incubated at 37°C with agitation (~120 rpm) for 7–10 days until the bacteria had reached the exponential growth phase (OD_600nm_ = 0.60–1.00), which is equivalent to 1 × 10^9^ bacteria/ml.

### THP-1 Cell Culture

THP-1 cell line (derived from a human acute monocyte leukemia; ATCC TIB-202) was cultured in complete RPMI-1640 (Gibco) (cRPMI) containing 10% (v/v) fetal bovine serum (FBS) (Sigma-Aldrich), 2 mM L-glutamine (Sigma-Aldrich), 100 U/ml penicillin (Sigma-Aldrich), 100 μg/ml streptomycin (Sigma-Aldrich) and 1 mM sodium pyruvate (Sigma-Aldrich) and left to grow in 5% v/v CO_2_ at 37°C for ~3 days before passaging. To count cells and assess cell death, equal volumes of cell suspension and Trypan Blue (0.4% w/v solution) were mixed and cells were counted using a haemocytometer with Neubauer rulings.

### Assay of rfBC Binding to *M. bovis* BCG

*M. bovis* BCG cells, grown to mid exponential growth phase, were harvested and washed in PBS and the concentration was adjusted to 1 × 10^7^ cells/ml in PBS. Then, 50 μl of *M. bovis* BCG bacterial suspension was dispensed into each well of a 96-well ELISA plate (Maxisorb™, Nunc). Plates were incubated at 37°C for 2 h, or 4°C overnight and washed once with PBS. Wells were then blocked for 2 h at 37°C with PBS containing 1% w/v of bovine serum albumin (BSA).

rfBC (100 μl/well) at various concentrations (0.65, 1.25, 2.5, 5, 10, 20, or 40 μg/ml) in buffer I (40 mM Tris-HCl, 200 mM NaCl, pH 7.4 containing 5 mM CaCl_2_ or 10 mM EDTA) were added to the coated wells and incubated for 1 h at 37°C followed by 1 h at 4°C. MBP was used a negative control. Microtiter wells were washed three times in buffer I. Bound proteins were detected by adding rabbit anti-bovine conglutinin polyclonal antibody or mouse anti-MBP monoclonal antibody (Sigma-Aldrich) to wells containing rfBC or MBP, respectively. Antibody dilutions were used as described above for Western Blotting. Antibodies were incubated for 1 h at 37°C, then wells were washed 3 times with buffer 1.

To detect the bound antibodies, Protein A-HRP (Sigma-Aldrich) or rabbit anti-mouse IgG-HRP conjugate were added to the wells at a dilution of 1:1,000 in PBS and incubated at 37°C for 1 h. Wells were then washed three times with buffer I. The substrate was added (100 μl/well of 3, 3′,5,5′-Tetramethylbenzidine; TMB) and the plate incubated in the dark for 1–5 min to allow color to develop, which was stopped by adding 50 μl of 0.5 M H_2_SO_4_. The optical density at 450 nm was then measured using (BioRad iMark microplate reader). All assays were conducted in triplicate.

### Maltose-Mediated Inhibition of rfBC Binding to *M. bovis* BCG

Microtitre plate wells were coated with *M. bovis* BCG cells and then blocked, as described above. 40 μg of rfBC was incubated with 0, 10, or 100 mM maltose in calcium buffer (40 mM Tris, 200 mM NaCl, 5 mM CaCl_2_ pH 7.4). Samples were incubated for 1 h at 37°C and, then for 1 h at 4°C. Binding of rfBC was detected as described above.

### Fluorescence Microscopy to Show Direct Binding of rfBC to *M. bovis* BCG

*M. bovis* BCG cells, grown to mid exponential growth phase, were harvested by centrifugation at 3,300 × g for 10 min and washed in PBS. The cell pellet containing 2 × 10^9^ bacteria was then re-suspended in 500 μl of buffer I with 5 mM CaCl_2_. rfBC (20 μg/ml) was then added and gently mixed via pipetting. As a negative control, a separate tube containing *M. bovis* BCG was incubated with MBP (20 μg/ml). Proteins were incubated with *M. bovis* BCG for 1 h at 37°C, followed by 1 h at 4°C with gentle shaking. Cells were then pelleted by centrifugation and washed twice in 500 μl of PBS. Rabbit anti-bovine conglutinin or mouse anti-MBP (dilutions as used for Western blotting) were added and incubated at 37°C for 1 h. Cells were washed 3 times in 500 μl PBS. Protein A conjugated with fluorescein isothiocyanate (FITC) (Sigma-Aldrich) (diluted 1:1,000 in PBS) was then added and incubated for 30 min in the dark at room temperature. Cells were washed 3 times in PBS and the cell pellet, resuspended in 20 μl of PBS, was spotted onto glass slides and covered with a coverslip. Slides were analyzed using a Leica fluorescence microscope (Leica DM4000).

### Direct Effect of rfBC on *M. bovis* BCG Growth

*M. bovis* BCG cells were grown to mid exponential growth phase and 2 ml culture were harvested as described above. The cell pellet containing 2 × 10^9^ bacteria was then resuspended in 1 ml of Middlebrook 7H9 with supplements (see above). Bacteria were separated into a single cell suspension by passing through a 1 ml syringe with 25-gauge needle 12–15 times to break clumps. Tubes containing 10-fold serial dilutions of the bacterial suspension (total of 1 ml) were then set up and incubated with 0, 10, 20, and 40 μg/ml of rfBC, in the presence of 5 mM CaCl_2_, for 1 h at 37°C, followed by 1 h at 4°C, with gentle shaking. Tubes were then vortexed and 200 μl of bacterial culture were plated on 7H10 plates, supplemented with 10% OADC and incubated for 2–3 weeks at 37°C. Bacterial colony forming units (CFU) were then counted by visual inspection. The experiment was performed in triplicate for each rfBC concentration and was repeated 3 times.

### Propidium Iodide Assay for *M. bovis* BCG Viability

*M. bovis* BCG cells were grown to mid exponential growth phase and 1 ml was harvested, as described above, into each of five microfuge tubes and washed in PBS. Cell pellets were then resuspended in buffer I with 5 mM CaCl_2_ and 0, 10, 20, and 40 μg/ml of rfBC added to each tube. All tubes were incubated for 1 h at 37°C in a shaker followed by one h at 4°C. Cells were then pelleted by centrifugation and resuspended in 1 ml of PBS. As a positive control, one additional rfBC-untreated sample was heated at 100°C for 10 min to lyse the cells. Propidium iodide (Sigma-Aldrich) was added to each tube (1 μg/ml), mixed by gentle pipetting and incubated for 20 min at room temperature in the dark. After incubation, tubes were centrifuged at 10,000 rpm for 10 min and washed with 500 μl of PBS three times. Cell pellets were then resuspended in 20 μl of PBS and the cell suspension spotted on a glass slides and covered with a coverslip. Slides were analyzed using a Leica fluorescence microscope (DM4000). The assay was performed in triplicate.

### Phagocytosis Analysis Using Colony Forming Units

THP-1 cells were seeded at 7 × 10^5^ cells/well in 12 well plates (Nunc) and were stimulated to adhere by adding 100 ng/ml of phorbol 12-myristate 13-acetate (PMA) (Sigma-Aldrich) and left to incubate for 48 h. Cells were then washed once with plain RPMI 1640 (serum free, antibiotic free) and then incubated overnight with fresh cRPMI. Cells were then washed once with plain RPMI 1640 and then incubated in 400 μl of plain RPMI 1640 for at least an hour before adding the bacteria.

*M. bovis* BCG cells were grown to mid exponential growth phase and 2 ml were harvested by centrifugation and washed in PBS. *M. bovis* BCG cells were then treated with or without human serum, prior to incubation with rfBC. For serum treatment, bacteria were resuspended in a 1/50 dilution of human serum in 200 μl of DGVB^++^ buffer (2.5 mM veronal buffer, pH 7.3, 72 mM NaCl, 140 mM glucose, 0.1% gelatin, 1 mM MgCl_2_, and 0.15 mM CaCl_2_) and incubated for 2 h at 37°C. Bacterial pellets were then collected by centrifugation and washed once with PBS. Complement activation by *M. bovis* BCG was confirmed using complement consumption assays as described previously (24).

The bacterial cell pellets (serum and non-serum treated) containing ~2 × 10^9^ bacteria were then resuspended in 1 ml plain RPMI 1640 with 5 mM CaCl_2_. Bacteria were separated into a single cell suspension by passing through a 1 ml syringe with 25-gauge needle 12–15 times to break clumps. Tubes containing the bacterial suspension were then set up and incubated with 0, 10, 20, and 40 μg/ml of rfBC, for 1 h at 37°C, followed by 1 h at 4°C, with gentle shaking.

Bacterial cell pellets were then collected via centrifugation and resuspended in 2 ml of fresh plain RPMI 1640. Then, 70 μl of bacterial suspension [equivalent to a multiplicity of infection (MOI) of 10:1, bacterial to macrophage] was added to each well containing the THP-1 cells. The medium was mixed by gently pipetting up and down 5 times. Plates were incubated at 37°C at 5% CO_2_ at 3, 6, 24, and 48 h timepoints.

After each time point, supernatants were collected from each well and stored at −20°C. Wells were washed 3 times with plain RPMI 1640 to remove any extracellular bacteria. THP-1 cells were removed using 1 ml of 0.25% trypsin and pelleted using centrifugation. For each time point, THP-1 cell pellets were then frozen at −80°C for RNA extraction and also analyzed for ingested mycobacteria as follows: THP-1 cells were lysed by re-suspending the cell pellets in 1 ml of 0.1% saponin followed by a series of vortexing for 10 min at room temperature. Plates containing Middlebrook 7H10 agar with supplements were prepared and four serial 1/10 dilutions of the cell lysate were made. Next, 10 μl of the concentrated mycobacterial suspension and diluted suspensions from each time-point were spotted onto the 7H10 agar plates. The plates were secured with parafilm and wrapped in aluminum foil, inverted and incubated at 37°C at 5% CO_2_ for 2–3 weeks. Bacterial CFU were then counted. Assay was performed in triplicate for each rfBC concentration and time point. Experiments were repeated three times.

### Phagocytosis Analysis Using Fluorescence Microscopy

THP-1 cells were seeded at 1 × 10^5^ cells on sterile coverslips (Sigma-Aldrich) in 12 well plates and were induced to adhere with PMA, incubated and washed, as described above. THP-1 cells were placed in 400 μl of plain RPMI 1640 for at least an hour before adding the bacteria. GFP-expressing *M. bovis* BCG was grown to mid exponential phase and harvested to obtain 1 × 10^9^ bacteria/ml. Mycobacteria were prepared as previously described for incubation with serum (complement deposition) and without serum. Bacterial pellets were dissolved in 200 μl of 7H9 medium with supplements and 5 mM CaCl_2_ and rfBC was added at final concentrations of 0, 10, 20, and 40 μg/ml. Samples were incubated at 37°C for 1 h followed by 1 h at 4°C with gentle shaking. Cells were then pelleted and resuspended in 2 ml of plain RPMI 1640. Next, 7 μl of the bacterial suspension from each of the rfBC concentrations was added to wells containing the THP-1 cells, giving a MOI of 10:1 bacteria to macrophages. Wells were mixed gently by pipetting up and down 5 times. Plates were incubated at 37°C and in 5% CO_2_ for 3 h. Supernatants were then discarded from wells and coverslips were washed 3 times with PBS. Cells were fixed in 4% paraformaldehyde at room temperature for 10 min and then washed a further three times in PBS. Cells were then stained with Hoechst (Thermo Scientific) (1:10,000 in PBS) and Alexafluor 546-conjugated wheat germ agglutinin (WGA) (Thermo Scientific) (1:500 in PBS) for 10 min at room temperature in the dark. Coverslips were then washed 5 times in PBS and mounted onto glass slides with 1 drop of anti-fade (Citifluor AF3) PBS solution.

To observe nuclear translocation of NF-κB, 1 × 10^5^ THP-1 cells per 13 mm coverslip were differentiated with PMA, and incubated with GFP-*M. bovis* BCG only, complement-deposited GFP-*M. bovis* BCG, GFP-*M. bovis* BCG pre-treated with 40 μg/ml of rfBC, or complement-deposited GFP-*M. bovis* BCG pre-treated with 40 μg/ml of rfBC. Samples were incubated in 500 μl of serum-free RPMI 1640 medium for 3 h. THP-1 cells only were used as a negative control. Following fixation, cells were permeabilized in 20 mM HEPES-NaOH pH 7.4, 300 mM sucrose, 50 mM NaCl, 3 mM MgCl_2_, 0.5% Triton X-100 and 10% sodium azide for 5 min on ice. Cells were then stained with rabbit anti-NF-κB p65 polyclonal antibodies (1:500, Santa Cruz Biotech), followed by Cy-3 goat anti-rabbit antibody (1:500, Sigma). Cells were mounted using Vectashield antifade with DAPI (Vector Labs) to reveal nuclei.

All Slides were observed under a Leica DM4000 fluorescence microscope at × 40 magnification. Images were processed using Image J software (https://imagej.nih.gov/ij/).

### Total RNA Preparation and cDNA Synthesis

THP-1 cell pellets were collected and lysed as described above. RNA extraction was performed using the GenElute Mammalian Total RNA Purification Kit (Sigma-Aldrich) according to the manufacturer's protocol. Samples were then treated with DNase (Sigma-Aldrich) to remove contaminating DNA according to the manufacturer's protocol. Samples were heated at 70°C for 10 min to inactivate DNase I and the RNase, and subsequently chilled on ice. The amount of RNA was measured using a NanoDrop 2000/2000c spectrophotometer (Thermo Fisher Scientific) at 260 nm and the ratio of absorbance at 260 nm and 280 nm was used to assess the purity of the RNA. cDNA was synthesized using High Capacity RNA to cDNA Kit (Applied Biosystems) following the manufacturer's protocol.

### qPCR Assay for Cytokine Expression and Data Analysis

The following oligonucleotide primers were used: 18S forward (5′-ATGGCCGTTCTTAGTTGGTG-3′) and 18 S reverse (5′-CGCTGAGCCAGTCAGTGTAG-3′); IL-1β forward (5′-GGACAAGCTGAGGAAGATGC-3′) and IL-1β reverse (5′-TCGTTATCCCATGTGTCGAA-3′); IL-6 forward (5′-GAAAGCAGCAAAGAGGCACT-3′) and IL-6 reverse (5′-TTTCACCAGGCAAGTCTCCT-3′); IL-10 forward (5′-TTACCTGGAGGAGGTGATGC-3′) and IL-10 reverse (5′-GGCCTTGCTCTTGTTTTCAC-3′); IL-12 forward (5′-AACTTGCAGCTGAAGCCATT-3′) and IL-12 reverse (5′-GACCTGAACGCAGAATGTCA-3′); TGF-β forward (5′-GTACCTGAACCCGTGTTGCT-3′) and TGF-β reverse (5′-GTATCGCCAGGAATTGTTGC-3′); TNF-α forward (5′-AGCCCATGTTGTAGCAAACC-3′) and TNF-α reverse (5′-TGAGGTACAGGCCCTCTGAT-3′). PCR was performed on all cDNA samples to assess the quality of the cDNA. The qPCR assay was performed for the expression of pro- and anti-inflammatory cytokines. The qPCR reaction consisted of 5 μl Power SYBR Green MasterMix (Applied Biosystems), 75 nM of forward and reverse primer and 500 ng template cDNA in a 10 μl final reaction volume. PCR was performed in a StepOne Plus Real-Time PCR System (Applied Biosystems). The initial steps were 2 min incubation at 50°C followed by 10 min incubation at 95°C, the template was then amplified for 40 cycles under these conditions: 15 s incubation at 95°C and 1 min incubation at 60°C. Samples were normalized using the expression of human 18 S rRNA. Data was analyzed using the StepOne software v2.3 (Applied Biosystems). Ct (cycle threshold) values for each cytokine target gene were calculated and the relative expression of each cytokine target gene was calculated using the Relative Quantification (RQ) value, using the formula: RQ = 2^−ΔΔ*Ct*^ and comparing relative expression with that of the 18 S rRNA constitutive gene product. Assays were conducted twice in triplicate.

### Multiplex Analysis

Supernatants were collected from the phagocytosis assays at 24 and 48 h to measure secreted cytokines (IL-6, IL-10, IL12p40, IL12p70, IL-1α, IL-1β, TNF-α, IL-13, IL-15, IL-17A, IL-9, TNF-β), chemokines (MCP-3, MDC, Eotaxin, Fractalkine, GRO, IL-8, IP-10, MCP-1, MIP-1α), growth factors (IL-2, EGF, FGF-2, G-CSF, GM-CSF, IL-3, IL-4, IL-5, IL-7, VEGF) and other ligands, cytokines and receptors (IFNA2, IFN-γ, FLT-3L, IL-1RA, sCD40L). MagPix Milliplex kit (EMD Millipore) was used to measure cytokine response following the manufacturer's protocol. 25 μL of assay buffer was added to each well of a 96-well plate, followed by the addition of 25 μL of standard, controls or supernatants of cells treated with *M. bovis* BCG in the presence or absence of rfBC and complement. 25 μL of magnetic beads coupled to analytes of interest were added in each well and incubated for 18 h at 4°C. The 96-well plate was washed with the assay buffer and 25 μL of detection antibodies were incubated with the beads for 1 h at room temperature. 25 μL of Streptavidin-Phycoerythrin was then added to each well and incubated for 30 min at room temperature with shaking at 750 rpm. Following a washing step, 150 μL of sheath fluid was added to each well and the plate was read using the Luminex Magpix instrument. Assays were conducted in duplicate.

### Statistical Analysis

Analysis of data for statistical significance was conducted using GraphPad Prism 6 for Windows (GraphPad Software, Inc.). Statistical analyses were made using a one-way ANOVA test. *P*-values < 0.05 were considered statistically significant, unless otherwise stated (ns, non-significant).

## Results

### Expression, Purification and Characterization of rfBC

**Figure 1:**
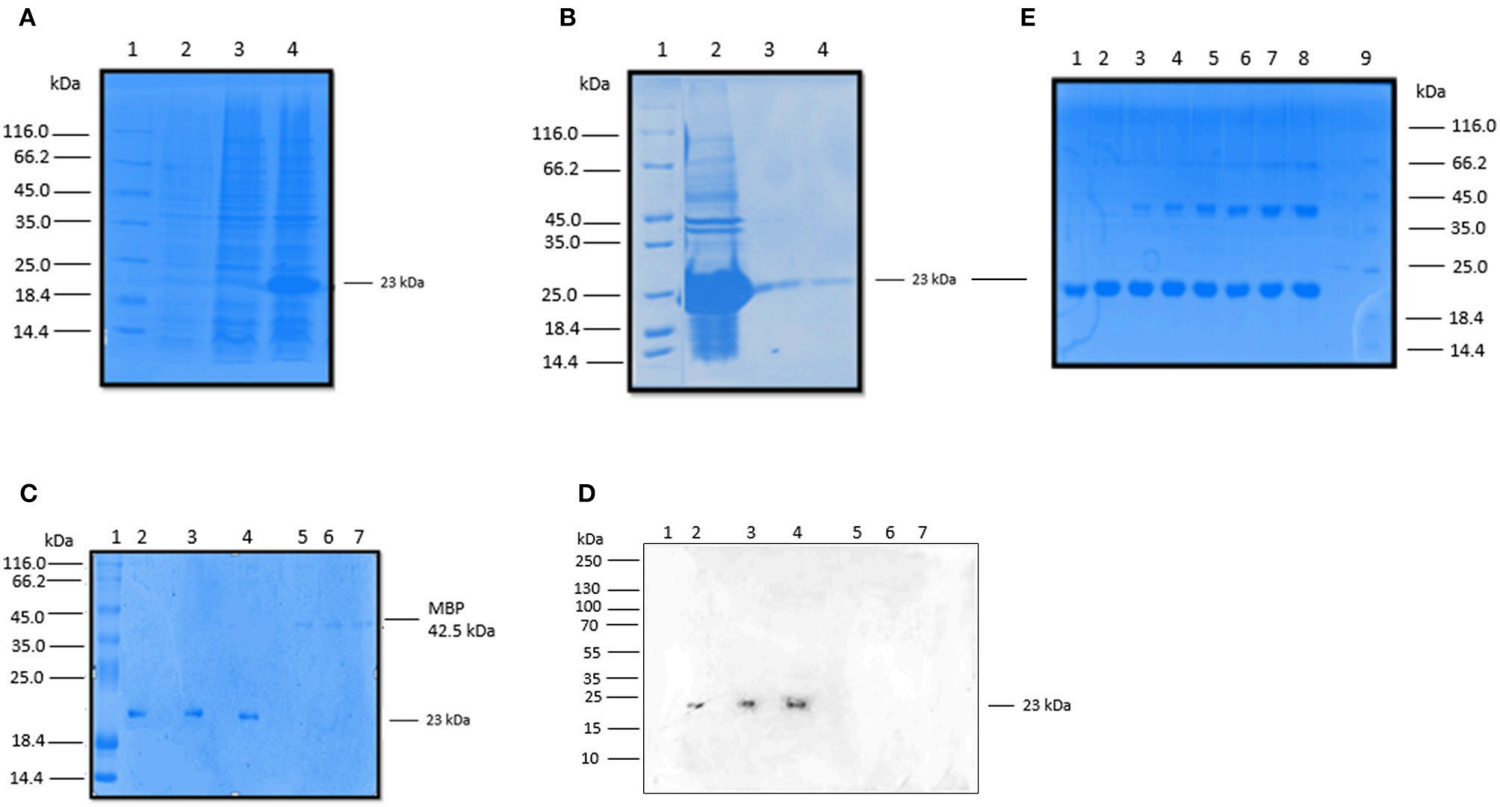
Expression and purification rfBC. **(A)** Recombinant bovine conglutinin (rfBC) containing the homotrimeric neck and CRD regions was expressed in *E. coli* BL21(λDE3) pLysS. 3 h after induction with 0.4 mM IPTG, rfBC accumulated as an over-expressed protein of ~23 kDa (lane 3) compared to uninduced cells (lane 2) and was observed as inclusion bodies (lane 4). Samples were resolved on a SDS-PAGE (12% w/v gel), followed by Coomassie staining. Protein marker (lane 1). **(B)** Purification of rfBC from inclusion bodies (lane 2) using denaturing and refolding in urea, followed by affinity chromatography on an agarose-N-acetylglucosamine column. The affinity-purified rfBC (Lane 3), was made LPS-free prior to use (lane 4). Samples were resolved on a SDS-PAGE (12% w/v gel), followed by Coomassie staining. Protein marker (lane 1). **(C)** Purified LPS-free rfBC verified by 12% SDS-PAGE, with 20 μg/ml of protein loaded in triplicate (lanes 2, 3, and 4), together with a negative control of 5 μg/ml of maltose binding protein (MBP) loaded also in triplicate (lanes 5, 6, and 7). Lane 1 is protein marker. **(D)** Western blot analysis confirms the identity and purity of rfBC (lanes 2, 3, and 4), using rabbit anti-bovine conglutinin. Western blotting with anti-MBP (not shown) confirmed no reaction with rfBC and reaction with 42.5 kDa MBP. **(E)** Chemical cross-linking of purified rfBC to show that it could form dimeric and trimeric structures. 20 μg/ml of rfBC were incubated with 1 mM BS^3^ [bis(sulfosuccinimidyl)suberate] for 0 min (lane 1), 1 min (lane 2), 2 min (lane 3), 4 min (lane 4), 8 min (Lane 5), 16 min (Lane 6), 32 min (lane 7), 64 min (lane 8). Protein marker (lane 9). Samples were resolved on a SDS-PAGE (12% w/v gel) followed by Coomassie staining and show dimers at 46 kDa and trimers at 69 kDa.

We expressed and purified a 154 amino acid long, recombinant fragment of conglutinin (rfBC), as previously described (13). rfBC is composed of the α-helical neck region (amino acids 197–224) and the C-terminal CRD region (amino acids 225–351) of the native mature protein. The protein was expressed successfully in *E. coli* (Figure 1A) and then purified from inclusion bodies yielding a band of 23 kDa on an SDS-PAGE gel (Figure 1B). Immunoblotting was performed using a rabbit anti-conglutinin antibody to confirm the identity of the 23 kDa band (Figures 1C,D). To further verify that rfBC was correctly folded and formed a homotrimer in solution, chemical cross-linking using BS^3^ was performed on the purified protein. rfBC formed trimers in a time- and BS^3^ concentration-dependant manner (Figure 1E).

### rfBC Binds to Mycobacteria

**Figure 2:**
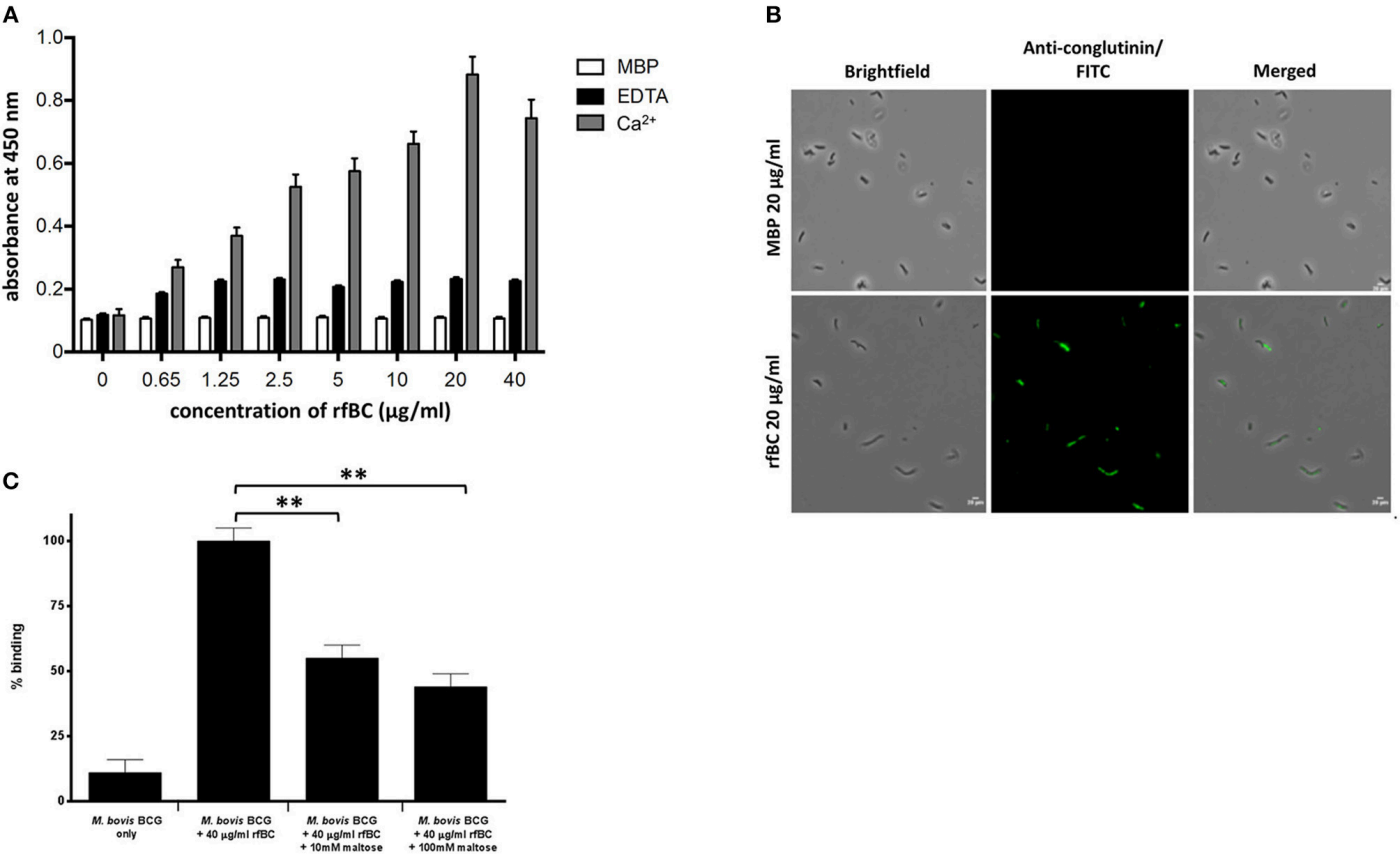
rfBC binds to *M. bovis* BCG. **(A)** rfBC binding to *M. bovis* BCG cells in the presence of Ca^2+^ with EDTA and maltose-binding protein used as negative controls. Two-fold serial dilutions of rfBC were performed in 40 mM Tris-HCl, 200 mM NaCl, pH 7.4 containing 5 mM CaCl_2_ or 10 mM EDTA and added to the mycobacteria and allowed to incubate for 2 h. This was followed by incubation with rabbit anti-bovine conglutinin antibody or mouse anti-MBP monoclonal antibody. Then, protein A-HRP was added and developed with the substrate 3, 3′,5,5′-Tetramethylbenzidine (TMB) and the color read at 450 nm. All assays were conducted in triplicate. **(B)** Direct binding of 20 μg/ml of rfBC to *M. bovis* BCG, with 20 μg/ml of MBP as a negative control. Cells were incubated for 2 h with either rfBC or MBP, followed by incubation with rabbit anti-bovine conglutinin antibody or mouse anti-MBP monoclonal antibody. Bacterial cells were then washed, fixed and stained with protein A conjugated with fluorescein isothiocyanate (FITC). Bar scale: 10 μm. **(C)** Competitive inhibition of rfBC binding to *M. bovis* BCG by maltose. rfBC was pre-treated with either 10 or 100 mM of maltose for 1 h before incubating with *M. bovis* BCG. Binding of rfBC was detected as described above. The percentage difference in rfBC binding for was calculated with respect to untreated 40 μg/ml of rfBC + *M. bovis* BCG (100%). Assay was conducted in triplicate. Bars denote ± 2 standard deviations. A one-way ANOVA test was performed on the data to determine significant differences in binding between *M. bovis* BCG + 40 μg/ml of rfBC (untreated) and 10 and 100 mM maltose. Both comparisons were significant (^**^p ≤ 0.01).

rfBC bound to *M. bovis* BCG in a calcium dependent manner (Figures 2A,B); binding in the presence of 5 mM EDTA was reduced 3- to 4-fold, whilst negligible binding was seen with MBP, used as a negative control protein (Figures 1A,B). The interaction between rfBC and *M. bovis* BCG reached saturation at 20 μg/ml of rfBC (Figures 2A,B); the concentration is comparable to serum levels of conglutinin in *B. taurus* of 12 μg/ml (4). Maltose, a glucose-glucose disaccharide analogous to mannose, was shown to inhibit the interaction of rfBC with M. bovis BCG, suggesting competitive inhibition of binding to surface motifs, such as LAM (Figure 2C). Furthermore, maltose has been shown to have similar inhibitory characteristics to mannose in our previous work (13). Maltose has previously been shown to inhibit the binding of SP-D to surface ligands (2–4).

### rfBC has a Direct Bacteriostatic Effect on Mycobacterial Growth

**Figure 3:**
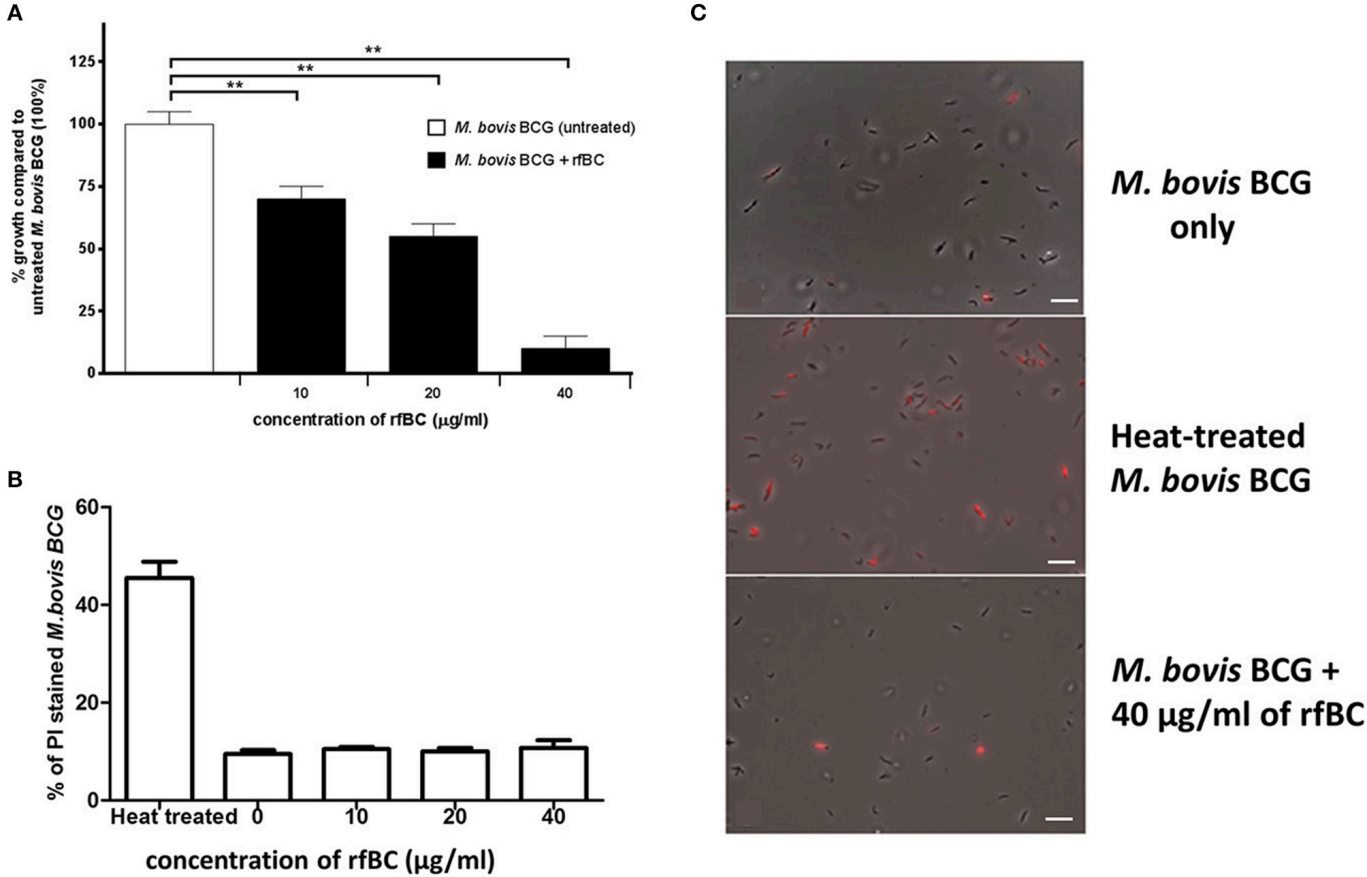
rfBC has a direct bacteriostatic effect on the growth of *M. bovis* BCG. **(A)** *M. bovis* BCG cells were grown in the presence of 10, 20, and 40 μg/ml of rfBC to assess any effect on bacterial growth. Untreated *M. bovis* BCG was used as a negative control. Bacterial colony forming units were obtained and counted. The percentage difference in growth for each rfBC concentration was calculated with respect to untreated *M. bovis* BCG as 100% (corresponding to 6 × 10^4^ cells). Bars denote ± 2 standard deviations. The experiment was performed in triplicate for each rfBC concentration and was repeated three times. A one-way ANOVA test was performed to determine significant differences in growth from untreated *M. bovis* BCG vs. treatment with rfBC at the different concentrations. All comparisons were significant (^**^p ≤ 0.01). **(B)** Propidium iodide (PI) assay was used to assess any bacterial cell lysis as a result of incubation with rfBC. *M. bovis* BCG cells were incubated for 2 h with 0, 10, 20, and 40 μg/ml of rfBC. Heat-treated *M. bovis* BCG was used as a positive control for cell lysis. PI was added, and cells spotted onto glass slides and analyzed using fluorescence microscopy. Fluorescent *M. bovis* BCG cells were counted from each sample and the total percentage of PI stained cells calculated for each rfBC concentration. Bars denote ± 2 standard deviations. The assay was performed in triplicate. A one-way ANOVA test was performed to determine significant differences in PI staining from heat-treated *M. bovis* BCG vs. treatment with rfBC at the different concentrations. All comparisons were significant (*p* ≤ 0.05), unless where shown (ns, not significant; *p* > 0.05). **(C)** Qualitative data showing PI staining of *M. bovis* BCG at 40 μg/ml of rfBC, compared to heat-treated *M. bovis* BCG and untreated *M. bovis* BCG. Bar scale 10 μm.

rfBC was observed to have a direct inhibitory effect on the *in vitro* growth of *M. bovis* BCG (Figure 3A). rfBC brought about significant reduction in *M. bovis* BCG growth that was 30% at 10 μg/ml and 90% at 40 μg/ml of rfBC, compared to rfBC-untreated *M. bovis* BCG (Figure 3A). This direct effect was also observed to be bacteriostatic in nature, rather than bactericidal, as virtually no bacterial cell lysis was observed on rfBC-treated *M. bovis* BCG, as determined by propidium iodide (PI) staining (Figures 3B,C). These results appear to suggest that conglutinin may be of importance in the control of systemic mycobacterial infection in *B. taurus*.

### rfBC Inhibits Uptake of *M. bovis* BCG by THP-1 Cells

**Figure 4:**
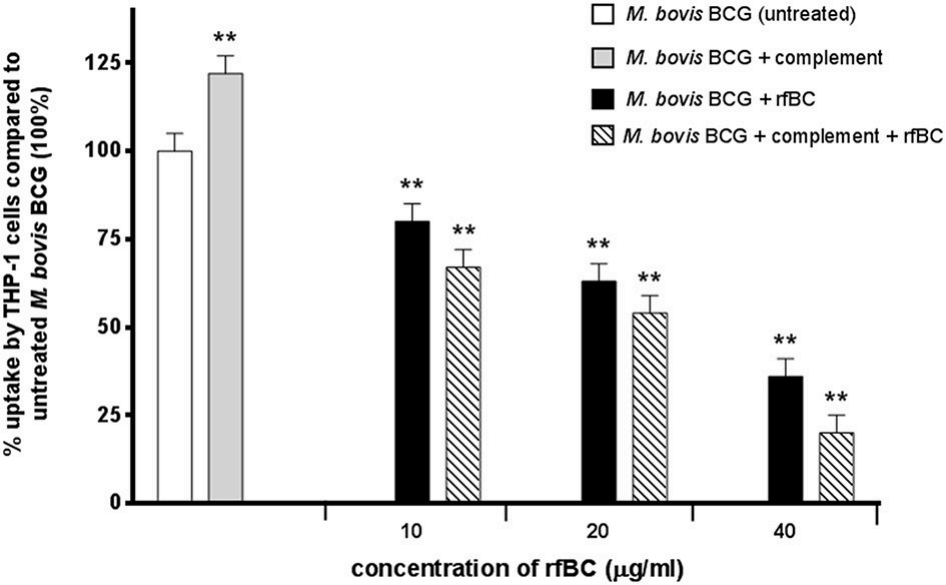
rfBC mediated inhibition of phagocytosis of *M. bovis* BCG by THP-1 cells, with and without complement deposition and its effect on the pro-inflammatory response. *M. bovis* BCG cells were pre-treated with human serum to induce complement deposition. *M. bovis* BCG cells with and without complement deposition were then treated with rfBC at concentrations of 0, 10, 20, and 40 μg/ml for 2 h. The *M. bovis* BCG cells were then incubated with THP-1 cells for 3 h. After THP-1 cell lysis, phagocytosed *M. bovis* BCG were measured by plating lysate on 7H10 media to obtain colony forming units (CFUs). The percentage difference in uptake of *M. bovis* BCG by THP-1 cells for each rfBC concentration was calculated with respect to untreated *M. bovis* BCG as 100% (corresponding to 4 × 10^4^ cells). Bars denote ± 2 standard deviations. The experiment was performed three times in triplicate. A one-way ANOVA test was performed on the data to determine significant differences in uptake from untreated *M. bovis* BCG vs. treatment with rfBC, complement only and complement plus rfBC. All comparisons were significant (^**^p ≤ 0.01).

**Figure 5:**
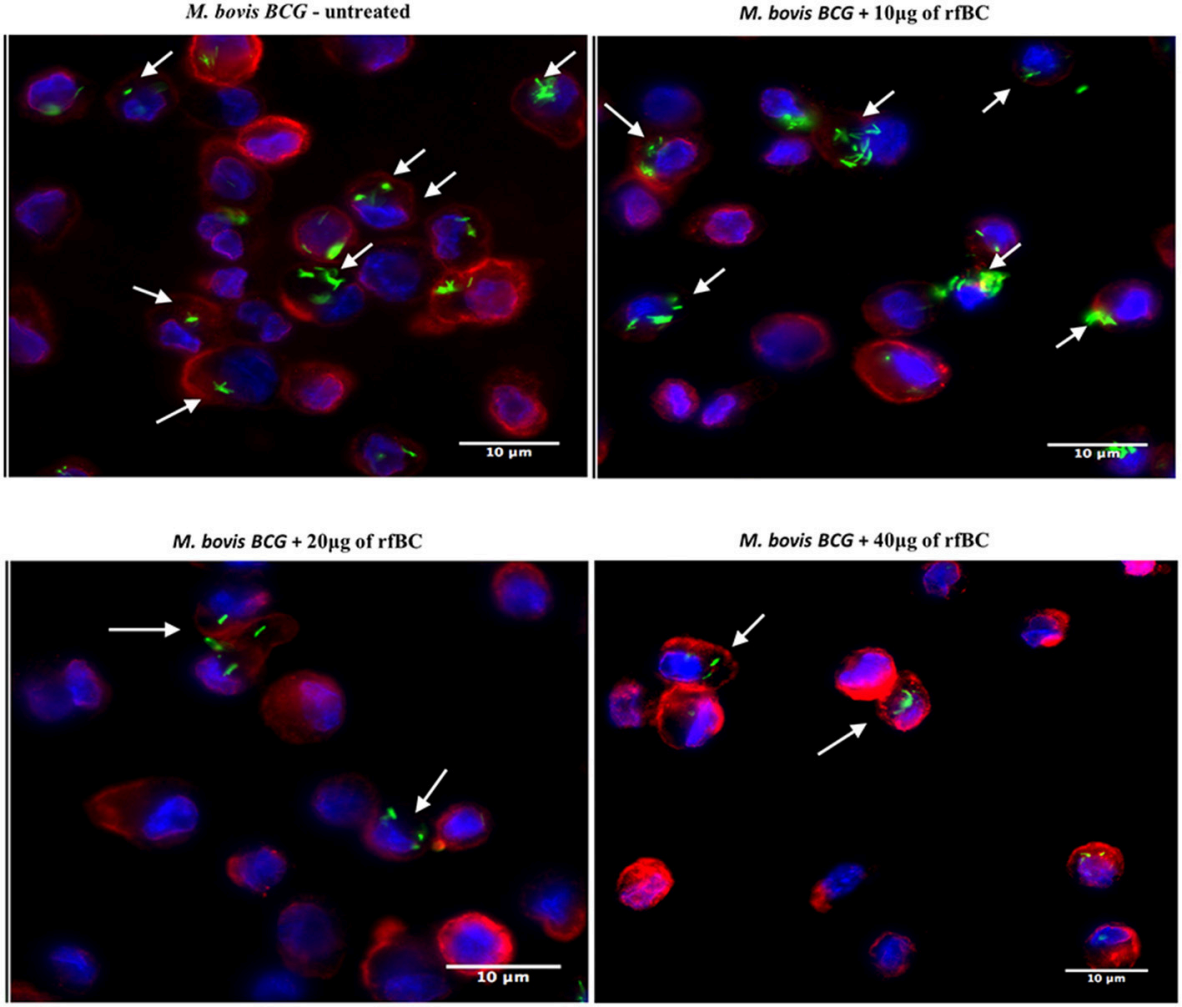
rfBC mediated inhibition of phagocytosis of *M. bovis* BCG by THP-1 macrophages. Differential phagocytosis of GFP-*M. bovis* BCG by THP-1 macrophages after treatment with 0, 10, 20, and 40 μg/ml of rfBC. Cells were incubated for 3 h. Cells were washed, fixed and stained with Alexafluor 546-conjugated wheat germ agglutinin to reveal the plasma membrane (red) and the nucleus was strained with Hoechst (blue). Images are shown as single sections; Arrows show foci of *M. bovis* BCG bacteria. bar scale 10 μm.

**Figure 6:**
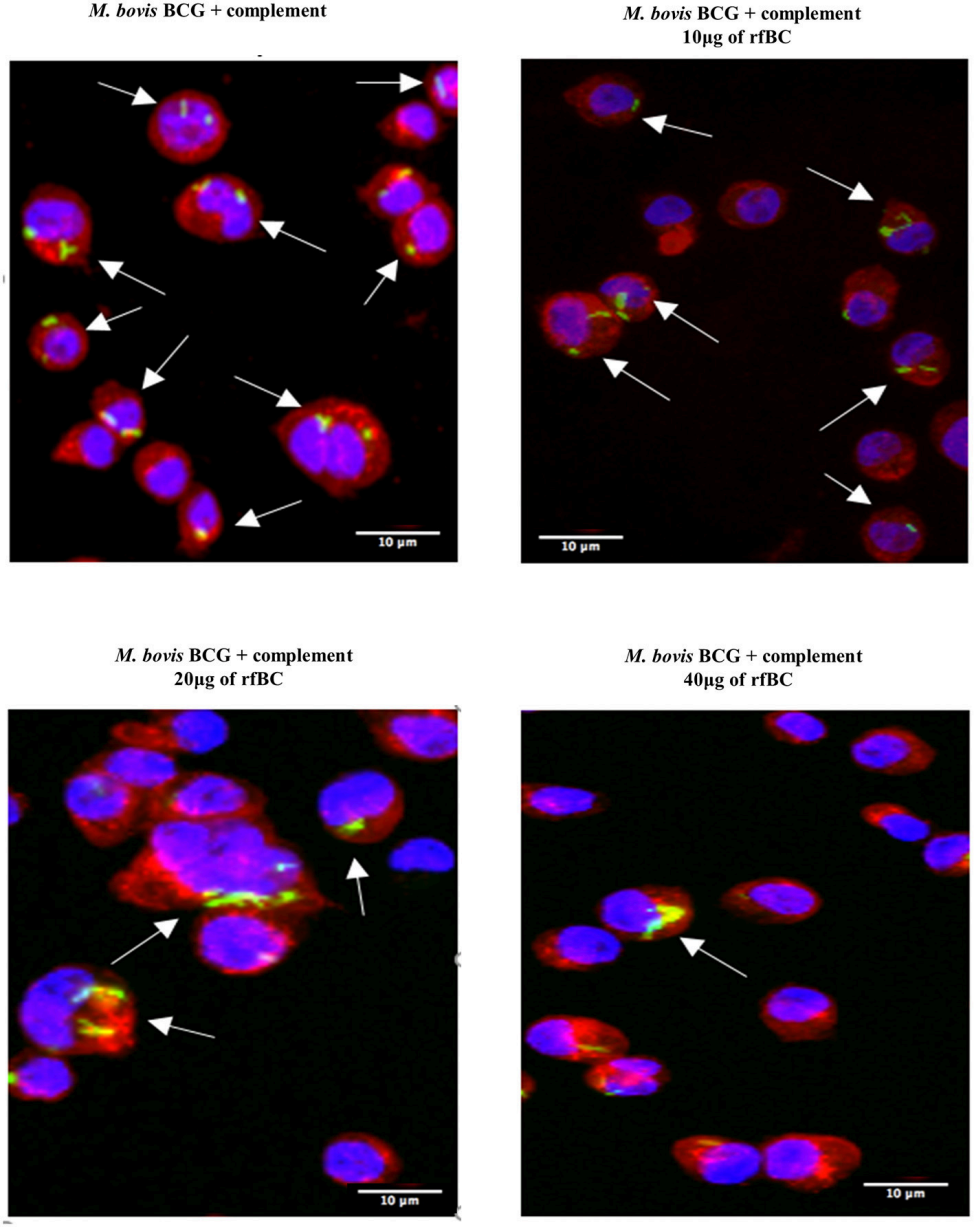
rfBC mediated inhibition of phagocytosis of complement-treated *M. bovis* BCG by THP-1. Differential phagocytosis of complement-treated GFP-*M. bovis* BCG by THP-1 macrophages after treatment with 0, 10, 20, and 40 μg/ml of rfBC. Cells were incubated for 3 h. Cells were washed, fixed and stained with Alexafluor 546-conjugated wheat germ agglutinin to reveal the plasma membrane (red) and the nucleus was strained with Hoechst (blue). Images are shown as single sections; Arrows show foci of *M. bovis* BCG bacteria. Bar scale 10 μm.

rfBC-treated *M. bovis* BCG showed reduced uptake by THP-1 cells, compared to untreated *M. bovis* BCG, with up to 65% inhibition (40 μg/ml of rfBC) (Figure 4). Since conglutinin is an iC3b-binding collectin, we also examined how it affected uptake of complement-treated *M. bovis* BCG. In this case, *M. bovis* BCG was incubated with human serum as a complement source, washed and then allowed to bind rfBC. Although complement treatment of *M. bovis* BCG resulted in enhanced phagocytosis (by ~ 22%) compared to untreated *M. bovis* BCG, we also observed marked inhibition in the phagocytosis of rfBC + complement-treated *M. bovis* BCG. This had a greater inhibitory effect than just treatment by rfBC alone (80% reduction in uptake at 40 μg/ml) (Figure 4). Fluorescence microscopy data also corroborated the inhibitory effect of rfBC on the phagocytosis of *M. bovis* BCG with and without complement deposition on the bacteria (Figures 5, 6). Thus, rfBC exhibits its uptake inhibitory effect both in the absence and presence of complement deposition.

### rfBC Dampens the Pro-Inflammatory Response During Phagocytosis

**Figure 7:**
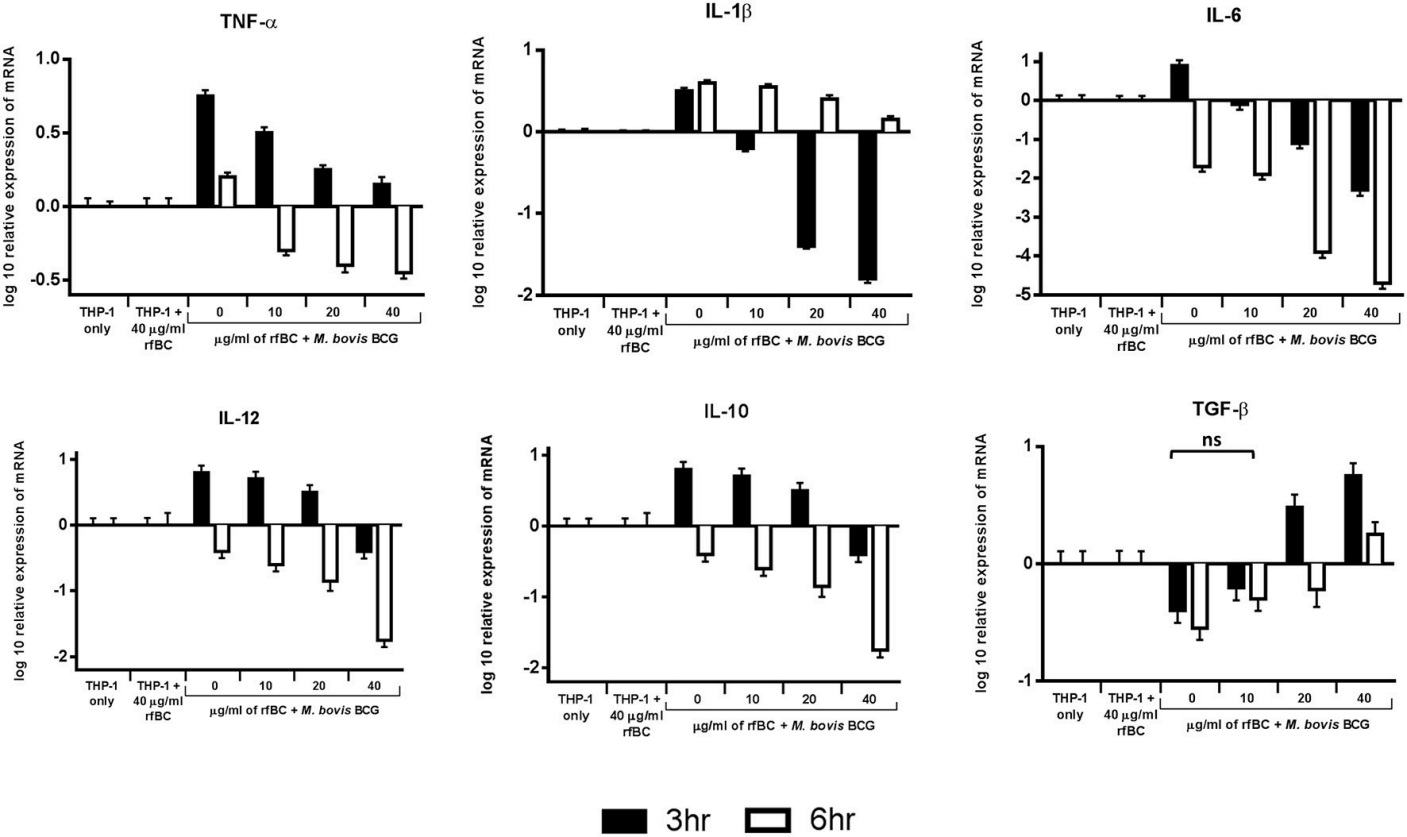
Temporal expression of cytokines by THP-1 cells incubated with *M. bovis* BCG and different concentrations of rfBC. qPCR analysis of the expression of cytokines (TNF-α, IL-1β, IL-6, IL-10, TGF-β, and IL-12) at 3 and 6 h incubation. The calibrator sample used was THP-1 cells only. The data was normalized to 18S rRNA gene expression which was used as an endogenous control. Assays were conducted in triplicate. The RQ value was calculated using the formula: RQ = 2^−ΔΔ*Ct*^. Error bars represent ± standard error of the mean. A 2-way ANOVA was performed on the data to compare differences in expression for each cytokine (e.g., between zero and different concentrations of rfBC added to *M. bovis* BCG). All comparisons are statistically significant (*p* ≤ 0.05), except where shown (ns, not significant).

We examined the early stages of the inflammatory response by measuring the mRNA expression levels of pro- and anti-inflammatory cytokines (Figure 7). These data show that rfBC-treated *M. bovis* BCG elicited a down-regulation of the pro-inflammatory cytokines (TNF-α, IL-1β, IL-6 and IL-12), at both 3 and 6 h of incubation with THP-1 macrophages. The effect on TNF-α is particularly dramatic. These levels in cytokine expression were compared to THP-1 cells only with no treatment with rfBC or *M. bovis* BCG only. In contrast, the anti-inflammatory cytokine TGF-β showed an increase in expression in the presence of rfBC-treated *M. bovis* BCG. However, the other anti-inflammatory cytokine, IL-10, was also down-regulated during these initial hours of interaction. THP-1 cells incubated only with rfBC did not show any significant changes in cytokine expression (Figure 7).

**Figure 8:**
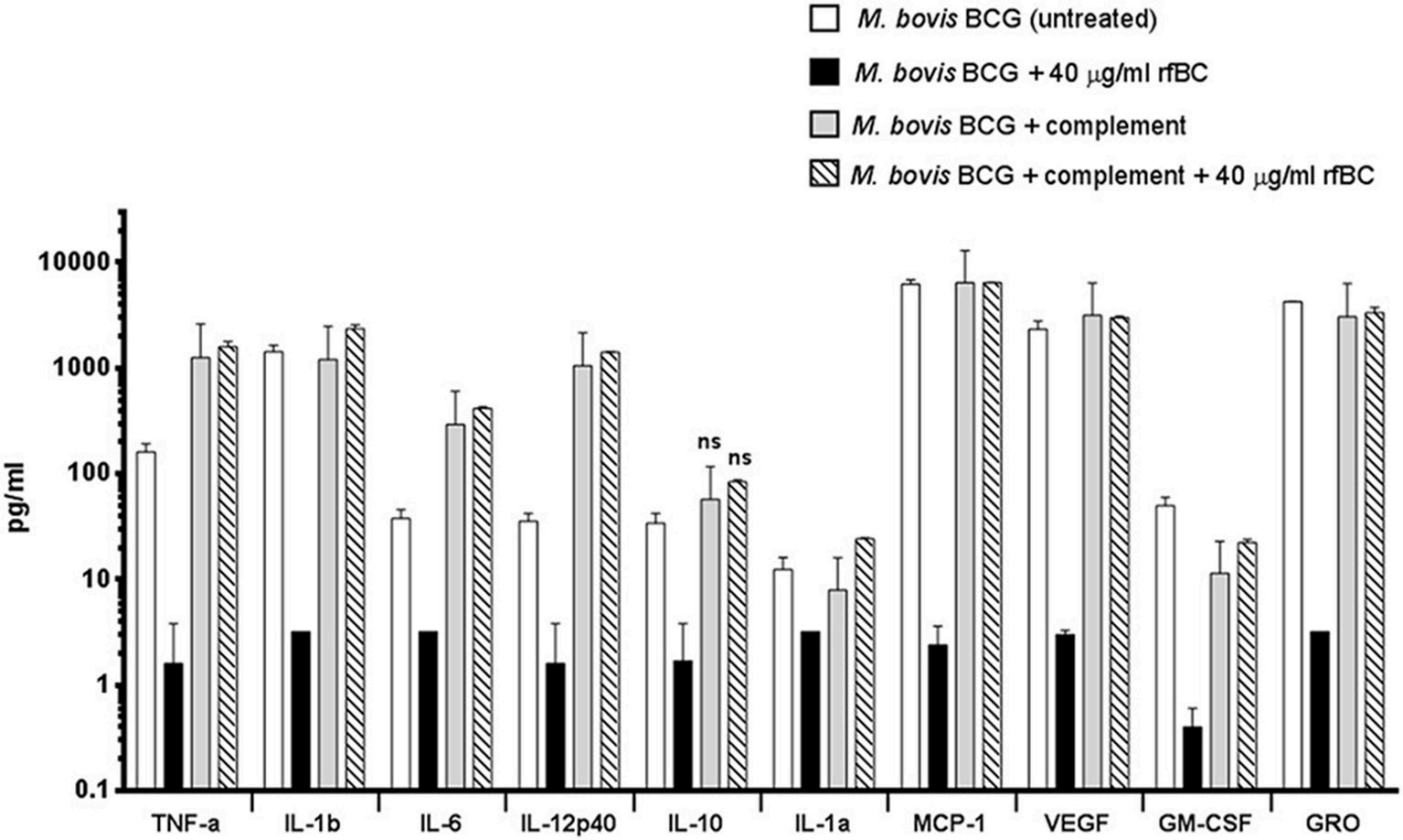
Multiplex cytokine analysis of supernatants collected at 24 h after phagocytosis of *M. bovis* BCG by macrophages incubated with rfBC and complement plus rfBC. Using multiplex analysis, the supernatants were measured for the levels of cytokines, chemokines and growth factors after 24 h of phagocytosis. Bars denote ± 2 standard deviations. Assays were performed in duplicate. A one-way ANOVA test was performed on the data to determine significant differences in cytokine/chemokine production after phagocytosis of untreated *M. bovis* BCG, vs. treatment with 40 μg/ml of rfBC, complement only and complement plus 40 μg/ml of rfBC. All comparisons were significant (^**^p ≤ 0.01), unless otherwise shown (ns: *p* > 0.05).

We also examined the immune response after 24 h of phagocytosis using multiplex cytokine array analysis to measure the protein levels of cytokines and chemokines produced by THP-1 cells in the supernatant. We observed that rfBC-treated *M. bovis* BCG triggered a broad decrease in the levels of pro-inflammatory cytokines (TNF-α, IL-1α, IL-1β, IL-6, IL-12p40), compared to untreated *M. bovis* BCG (Figure 8). A similar decrease in levels was also observed for the chemotractant MCP-1 and growth factors (VEGF, GM-CSF, and GRO), further suggesting a dampening of pro-inflammatory responses. The decrease in levels of IL-12p40 by rfBC also points to a possible suppression of Th1 cytokines that can be produced by CD4^+^ T-cells, leading to a reduction in IFN-γ which is also critical for sustained intracellular killing by macrophages and granuloma formation. The decrease of IL-10 by rfBC suggests the suppression of anti-mycobacterial responses by the macrophage.

**Figure 9:**
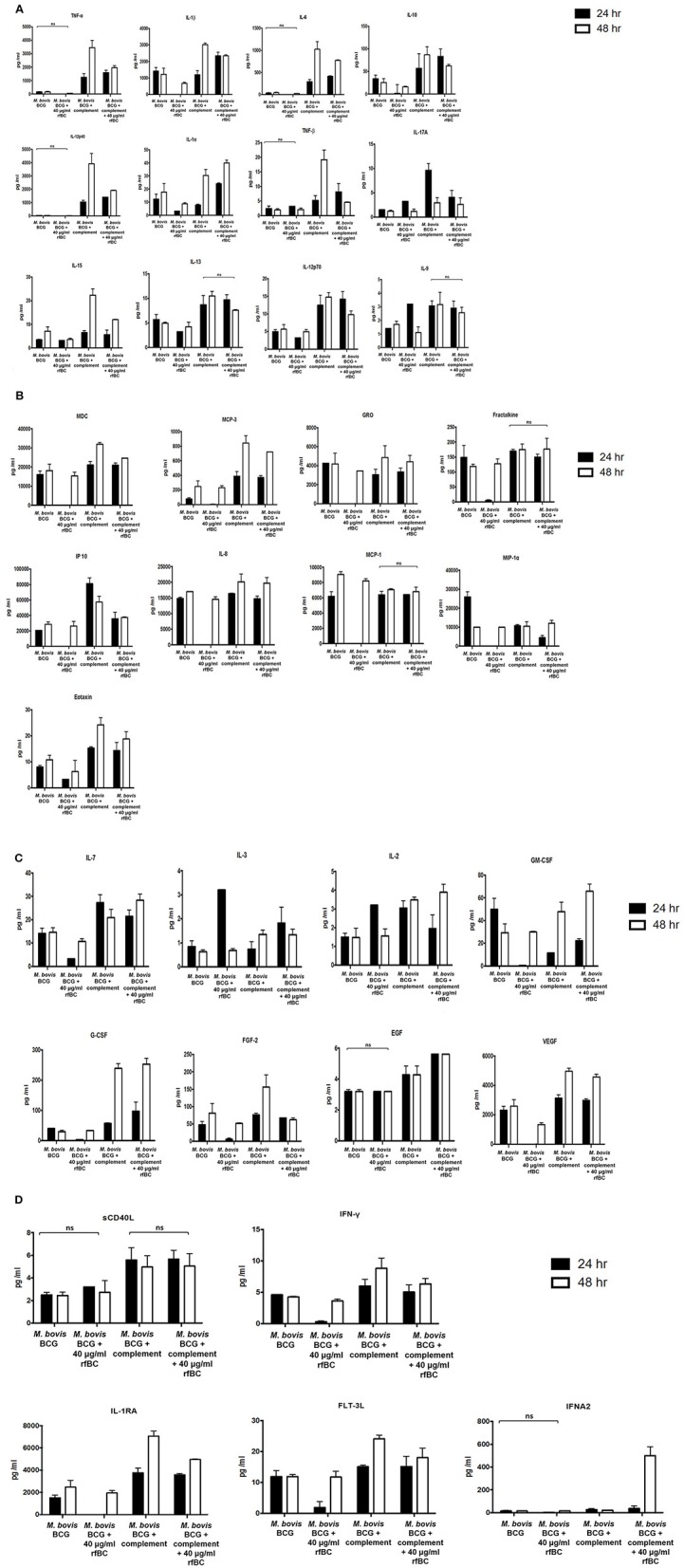
Multiplex cytokine analysis of supernatants collected at 24 and 48 h after phagocytosis of *M. bovis* BCG by macrophages incubated with rfBC and complement plus rfBC. The supernatants were collected from phagocytosis assay of *M. bovis BCG* in the presence or absence of rfBC and complement plus rfBC at 24 h and 48 h time points. The levels of cytokine production was measured for **(A)** (IL-6, IL-10, IL12p40, IL12p70, IL-1α, IL-1β, TNF-α, IL-13, IL-15, IL-17A, IL-9, TNF-β), **(B)** chemokines (MCP-3, MDC, Eotaxin, Fractalkine, GRO, IL-8, IP-10, MCP-1, MIP-1α), **(C)** growth factors (IL-2, EGF, FGF-2, G-CSF, GM-CSF, IL-3, IL-7, VEGF), and **(D)** related ligands and receptors (IFNA2, IFN-γ, FLT-3L, IL-1RA, sCD40L) using multiplex analysis. Error bars represent standard deviation. A one-way ANOVA test was performed on the data to determine significant differences in expression of cytokine production from untreated *M. bovis* BCG vs. treatment with rfBC, and *M. bovis* BCG treated with complement vs. treatment with complement plus rfBC. All comparisons were significant (*p* ≤ 0.05), unless where shown (ns, not significant; *p* > 0.05). Supernatants were analyzed in duplicate.

The increase in uptake of complement-treated *M. bovis* BCG compared to untreated *M. bovis* BCG (Figure 4), coincided with an enhancement of the pro-inflammatory response (Figures 8, 9A–D). In particular, TNF-α, IL-1β, IL-6, and IL-12p40 were significantly elevated. In contrast, for the anti-inflammatory cytokine IL-10, no significant change in levels was observed. This observed enhancement of the pro-inflammatory response was also maintained during the uptake of rfBC+complement-treated *M. bovis* BCG (Figure 8).

The increase in pro-inflammatory cytokines (e.g., TNF-α) after the initial stages of phagocytosis of mycobacteria by macrophages is thought to be particularly crucial for the killing of intracellular mycobacteria and for the formation of the protective granuloma and is observed in the untreated *M. bovis* BCG (Figure 8). Furthermore, TNF-α production was enhanced further in complement-deposited *M. bovis* BCG (Figure 8). Intriguingly, rfBC does not seem to have a noticeable effect on the inflammatory response of complement-deposited *M. bovis* BCG (i.e., *M. bovis* BCG + complement + rfBC), suggesting that any phagocytosis of *M. bovis* BCG that has occurred may be exclusively via complement receptors and this may be sufficient to mount an optimum inflammatory response.

**Figure 10:**
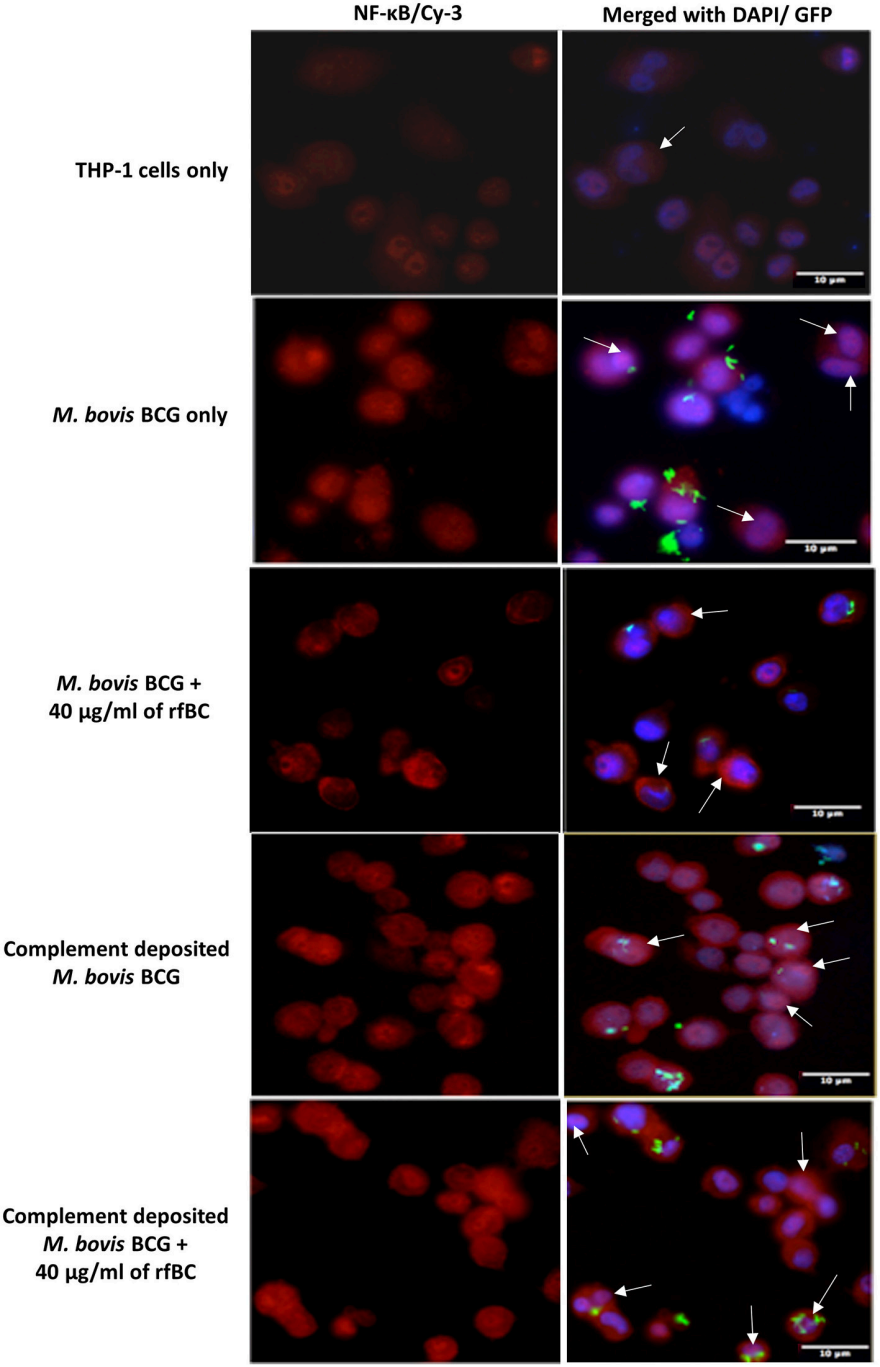
Cytoplasm to nucleus translocation of NF-κB in THP-1 cells co-cultured with *M. bovis* BCG with or without rfBC/complement deposition. THP-1 cells were incubated with GFP-*M. bovis* BCG pre-treated with 40 μg/ml of rfBC, with and without complement deposition. These were compared to GFP-*M. bovis* BCG only and complement-deposited GFP-*M. bovis* BCG only. THP-1 cells only were used as negative controls. Bacteria and THP-1 cells were incubated for 3 h for phagocytosis to occur. Cells were washed, fixed, permeabilised and incubated with rabbit anti-NF-κB p65 antibodies, followed by Cy3-conjugated goal anti-rabbit antibody (red). The nucleus was stained with DAPI (blue). Arrows highlight nuclear NF-κB in the merged images. Scale bar 20 μm.

Further evidence in support of dampening of the pro-inflammatory response is shown by the NF-κB nuclear translocation in response to complement-deposited *M. bovis* BCG, showing an increase in the levels of pro-inflammatory cytokines, which seems to be restricted in response to rfBC + complement bound *M. bovis* BCG, (Figure 10). Since NF-κB is a key transcription factor that regulates the expression of a number of pro-inflammatory cytokines, we studied the translocation of NF-κB in THP-1 cells stained with an antibody against the p65 subunit of NF-κB and performed analysis using fluorescence microscopy. We observed that NF-κB translocation was more pronounced in complement-treated *M. bovis* BCG and rfBC + complement-treated *M. bovis* BCG, when compared to the control (THP-1 cells only) (Figure 10). Similar levels of NF-κB translocation were also observed in untreated *M. bovis* BCG. However, virtually no translocation was observed for rfBC-treated *M. bovis* BCG, thus explaining the downregulation of pro-inflammatory cytokines (TNF-α, IL-1β, and IL-6) and chemokines (IL-8, MCP-1, and IP-10) (Figures 4, 9A,B).

We also observed that the ability of rfBC to suppress pro-inflammatory cytokines appeared to be transient as it recovered somewhat after 48 h, to levels observed for untreated *M. bovis* BCG (Figures 9A–D). A number of other secreted cytokines, chemokines, growth factors, ligands and receptors were also examined which suggested an anti-inflammatory effect by rfBC (Figures 9A–D). Coupled together, these data suggest that conglutinin has an anti-inflammatory effect that may suppress anti-mycobacterial immunity and promote the extracellular persistence of the *M. bovis* pathogen in *B. taurus*.

## Discussion

In the present study, we have studied the intriguing bovine collectin, conglutinin, and its possible role in tuberculosis host-pathogen interactions. We have used *M. bovis* BCG as a model for *M. bovis* and *M. tuberculosis* and demonstrate that a homotrimeric recombinant truncated form of conglutinin (rfBC), composed of the α-helical neck region and the CRD (13), was able to bind to *M. bovis* BCG and had a major influence on its phagocytosis by THP-1 macrophages. We also show that this interaction can occur with and without complement deposition on the bacterium, although complement treatment profoundly altered the host's inflammatory response. rfBC significantly inhibited the uptake of *M. bovis* BCG by THP-1 cells and this occurred in a dose-dependent manner. In contrast, complement deposition enhanced uptake of *M. bovis* BCG by THP-1 macrophages, but when these mycobacteria were treated with rfBC, significant inhibition of uptake was also observed. Our recent studies have also highlighted similar inhibitory effects by complement regulatory proteins (factor H and properdin) on the phagocytosis of *M. bovis* BCG by THP-1 macrophages (34, 35). These observations suggest that conglutinin is masking mycobacterial ligands and inhibiting uptake, possibly through mannose receptors, but also is masking interactions with complement by binding to iC3b and inhibiting uptake though complement receptors (e.g., CR3). These data are the first to show an anti-opsonic effect of conglutinin on mycobacteria and that this can occur in a complement dependent and independent manner.

Conglutinin is a member of the collectin protein family which are soluble PRRs that are able to bind to oligosaccharide and glycolipid structures (PAMPs) on the surface of microbes. Conglutinin is known to have antimicrobial properties, being able to opsonise and inhibit haemagglutination of influenza A virus and binds to rotavirus, *E. coli* and *S. typhimurium* as well as LPS (8, 10–13). However, conglutinin's role in bovine tuberculosis has not been extensively explored. This is of particular importance since conglutinin is found in *Bovidae* species (e.g., cattle: *B. taurus*) (2). Bovine tuberculosis (*M. bovis* infection) remains not just a significant agricultural economic problem in the UK and worldwide, but is also an important zoonosis affecting a wide range of domestic and wild animals, and including humans (20–23). We observed that rfBC bound to *M. bovis* BCG and that this binding was dose dependent, reaching saturation of binding at 20 μg/ml of rfBC, which is similar to the serum levels of conglutinin observed in cattle (4). Binding of rfBC to *M. bovis* BCG was observed to be greater in the presence of Ca^2+^ than in the presence of EDTA. This is the first definitive observation of conglutinin pattern recognition and binding to a Gram-positive bacterium (e.g., mycobacteria). SP-D has also recently been shown to bind to *M. bovis BCG* (36). The mycobacterial ligand for conglutinin is likely to be those reported for SP-D and *M. tuberculosis*, namely the terminal dimannosyl units of lipoarabinomannan (LAM) (37, 38), since SP-D is structurally similar to conglutinin (2) and *M. bovis* BCG shares an almost identical bacterial surface, including LAMs (39, 40). We observed that rfBC binding to *M. bovis* BCG was inhibited when rfBC was pre-treated with maltose. Maltose has been shown to be an effective inhibitor of conglutinin and rfBC binding to surface ligands (2, 13). The results show that maltose was able to block the CRD region of rfBC preventing interaction with sugar moieties, such as LAM on the surface of the mycobacterium. These observations are in agreement with results from our previous work that showed various sugars were also able to bind to the CRD region of rfBC and that these specificities are similar for full length conglutinin (2, 13).

We also observed that rfBC could directly inhibit the growth of *M. bovis* BCG *in vitro* in a dose-dependent manner. The mechanism of inhibition is not known, but it is clear that this inhibition is bacteriostatic, rather bacteriocidal (e.g., no bacterial cell lysis was observed in rfBC treated mycobacteria). To our knowledge, this is the first demonstration of a direct bacteriostatic effect by a collectin on a Gram-positive bacterium. Previous studies have shown growth inhibition in Gram-negative bacteria by SP-A and SP-D by increased membrane permeability, but this was also not bacteriocidal (41–43). No evidence of agglutination of *M. bovis* BCG was observed by rfBC. This is because, unlike the native conglutinin dodecameric form which is multivalent with respect to the trimeric CRDs, rfBC is only trivalent with respect to CRDs and lacks the long rigid collagen-like region so is unable to form the cruciform structure that allows for greater bridging interactions between the bacteria. These observations support similar findings on the aggregation properties of SP-D and its truncated recombinant form rhfSP-D (32, 44).

Our data also shows that conglutinin does not enhance interactions of mycobacteria with macrophages, but actually plays a role in reducing this interaction. This observation has also been described for SP-D and mycobacteria and this reduced interaction is also independent of any bacterial agglutination (32). Similar to SP-D (32), we found conglutinin to be able to significantly inhibit the uptake of mycobacteria by macrophages

(THP-1 cells), possibly by altering the usual interaction between the ligands on the mycobacterial surface and receptors on the macrophage. The main routes for *M. tuberculosis* phagocytosis by macrophages is via the mannose receptor and complement receptors (26, 45–47). Based on our study, we consider that conglutinin may intefere with both these interactions. Firstly, like SP-D, it is likely that conglutinin binds to the terminal mannosyl units of LAM on *M. bovis* BCG, which are ligands for the mannose receptor, and that this interaction is reducing the uptake of *M. bovis* BCG by conglutinin binding to LAM on the bacterial surface and blocking its interaction with the mannose receptor on macrophages. We have indeed observed a marked inhibition by rfBC of *M. bovis* BCG phagocytosis by THP-1 macrophages and this is dose-dependent and the inhibitory effect is greater than that observed for SP-D (32). Secondly, we investigated the effect of complement and how conglutinin may influence the interaction between mycobacteria and macrophages. Previous studies have shown the deposition of complement component C3 on mycobacteria and its role in the uptake of the pathogen via complement receptors CR1, CR3, and CR4 on the macrophage (26–31). C3 binding to *M. tuberculosis* and *M. bovis* BCG has been shown to occur via the three complement pathways and C3 is present on the bacteria in the forms C3b and iC3b (24, 27). C3b is necessary for the complement cascade to progress toward the terminal membrane attack complex (MAC), but iC3b is not and cannot take part in this reaction. Furthermore, C3b is a ligand for CR1, whilst iC3b is a ligand for CR3 and CR4 (48). iC3b is formed on the surface of microbes from C3b, by cleavage by factor I together with cofactors like factor H and CR1 (49). We have observed that complement-deposited *M. bovis* BCG are inhibited from phagocytosis by THP-1 macrophages by rfBC. Conglutinin is a known unique binder of iC3b, as it has a selective affinity for the mannose oligosccharides in the α-chain of iC3b (14). Thus, a likely mechanism of this inhibition is the binding of conglutinin to iC3b deposited on the bacterial surface, blocking phagocytosis via macrophage receptors CR3 and CR4. In contrast, we observed an enhancement of phagocytosis by complement-deposited *M. bovis* BCG by THP-1 macrophages without the presence of rfBC, compared to *M. bovis* BCG only, demonstrating likely enhanced uptake by CR1, CR3, and CR4. Consequently, conglutinin significantly inhibits uptake of *M. bovis* BCG by macrophages in a complement dependent and complement-independent manner, blocking host-bacterial interactions via complement receptors and mannose receptor, respectively. To our knowledge, this is the first reported observation of mycobacterial phagocytosis being blocked via two distinct mechanisms by a single collectin.

To investigate the role of conglutinin further in mycobacterial infection, we examined the subsequent immune response by THP-1 macrophages during phagocytosis. We observed that *M. bovis* BCG, treated with rfBC, dampened pro-inflammatory response after 24 h of phagocytosis, compared to *M. bovis* BCG only, after a significant inhibition of bacterial uptake by the macrophages. The pro-infammatory cytokine/chemokines levels at 48 h were similar to levels observed for *M. bovis BCG* only. In contrast, the pro-inflammatory response was elevated significantly in complement-deposited *M. bovis* BCG and complement-deposited *M. bovis* BCG treated with rfBC and were at similar levels to *M. bovis BCG* only. However, for complement-deposited *M. bovis* BCG treated with rfBC, there was significant inhibition of phagocytosis, whilst for complement-deposited *M. bovis* BCG there was an enhancement of uptake.

In the bovine lung, it is not clear whether there are significant local levels of conglutinin. One study has shown conglutinin in lung macrophages, being localized to the phagolysosome, but it is unclear whether this was internalized as a result of phagocytosis, or synthesized locally, or if it is membrane bound (50). SP-A and SP-D are also the main collectins found in the lungs and have potent anti-microbial properties (17). However, if conglutinin is present in the bovine lung, then recruitment of conglutinin by mycobacteria may be important in the initial stages of tuberculosis infection. After inhalation, among the first host cells *M. bovis* encounters is the alveolar macrophage and conglutinin inhibition of uptake of the bacterium by macrophages may be protective. *In vivo*, alveolar macrophages phagocytose mycobacteria, but ultimately fail to destroy them and provide a niche for the bacteria to survive and persist. Conglutinin may inhibit this and facilitate the exclusion of the pathogen to an extracellular environment enhancing microbial clearence e.g., ciliary mucosa. The dampening of the pro-inflammatory response by conglutinin may also supress the production of important cytokines and chemoattractants (TNF-α, IL-1β, IL-6, IL-12p40) that recuit inflammatory cells and discourage inflammation and tissue remodeling necessary for granuloma formation (51–54). In the absence of complement in the lungs, this may be a preferred stratergy by the host to protect against mycobacterial infection. However, there is evidence of complement in the lungs that could support C3 deposition on mycobacteria (27). In this scenerio, conglutinin could be acting once again to maintain the pathogen in the extracellular milieu, while also simultaneously tailoring the enhanced pro-inflammatory response to the extracellular environment for clearance of the pathogen.

Conglutinin is primarily found in serum in the bovine host and it is in this environment that it may play a crucial role. Conglutinin is synthesized in the liver and secreted into the blood plasma at a mean serum concentration of 12 μg/ml (4), which is similar to the molar concentrations of rfBC used in our study. Interestingly, it has also been shown that low plasma levels of conglutinin in cattle predispose to respiratory infection and the basis for this was found to be genetic in some breeds (4). *M. bovis* bacteremia in cattle appears to be rare with only a few cases being reported (55–57). This may be due to an under-reporting of such cases (58), or may be due to enhanced ability of the bovine host to cope with disseminated infection and bacteremia. It is plausible that conglutinin plays a major role in controlling *M. bovis* bacteremia, since it is present in significant amounts in the serum and low levels are linked with susceptibility to infection (4). These reports on *M. bovis* bacteremia and the findings from our study, point to an intriguing possibility that conglutinin is involved, together with complement, in controlling the dissemination of *M. bovis* infection.

The macrophage is a natural environment for virulent mycobacteria, but for many other pathogens entry into this host cell is terminal. Factors that enhance phagocytosis of mycobacteria into macrophages are likely to be harmful to the host. We envisage that reducing macrophage phagocytosis and growth of mycobacteria (*M. bovis* and *M. tuberculosis*) will be protective for the host. Therefore, findings from our study suggest that conglutinin plays an important host defense role against bovine tuberculosis infection by inhibiting phagocytosis of mycobacteria by macrophages via two key known mechanisms of interaction, i.e., the macrophage mannose receptor and complement iC3b receptors. Conglutinin probably enhances the clearance of mycobacterial infection by downplaying pathogen immune evasion strategies and also augmenting the pro-inflammatory response against infection by down-regulating the cellular immune response against infection, supressing tissue damage and steering toward the extracellular clearance of the pathogen. This study provides insights into previously unknown involvement of conglutinin and mycobacterial infection. Further studies using pathogenic *M. bovis* and *M. tuberculosis in vitro* (e.g., bovine macrophages) and *in vivo* are needed to fully characterize the nature and consequences of involvement of conglutinin in tuberculosis pathogenesis.

## Author Contributions

AM and AT carried out most of the experiments with help from LK and AK. UH, RS, and MA-A provided crucial reagents. AT and UK designed experiments and wrote the manuscript.

### Conflict of Interest Statement

The authors declare that the research was conducted in the absence of any commercial or financial relationships that could be construed as a potential conflict of interest.

## Abbreviations

TB: tuberculosis
BCG: Bacillus Calmette-Guerin
CR: complement receptors
MAC: membrane attack complex
PRR: pattern recognition receptors
PAMP: pathogen-associated molecular patterns
BC: bovine conglutinin.
